# The MYBL2–CCL2 axis promotes tumor progression and resistance to anti-PD-1 therapy in ovarian cancer by inducing immunosuppressive macrophages

**DOI:** 10.1186/s12935-023-03079-2

**Published:** 2023-10-21

**Authors:** Baoyue Pan, Ting Wan, Yun Zhou, Shuting Huang, Linjing Yuan, Yinan Jiang, Xiaojing Zheng, Pingping Liu, Huiling Xiang, Mingxiu Ju, Rongzhen Luo, Weihua Jia, ChunYan Lan, Jundong Li, Min Zheng

**Affiliations:** 1 Department of Gynecology, State Key Laboratory of Oncology in South China, Collaborative Innovation Center for Cancer Medicine, Sun Yat-sen University Cancer Center, Guangzhou, 510060 China; 2 Department of Gynecology, Guangdong Provincial People’s Hospital, Guangdong Academy of Medical Sciences, Guangzhou, 510080 China; 3 Department of Gynecology, The First Affiliated Hospital of Sun Yat-sen University, Guangzhou, 510080 China; 4 Department of Pathology, State Key Laboratory of Oncology in South China, Collaborative Innovation Center for Cancer Medicine, Sun Yat-Sen University Cancer Center, Guangzhou, 510060 China; 5 State Key Laboratory of Oncology in South China, Collaborative Innovation Center for Cancer Medicine, Biobank of Sun Yat-sen University Cancer Center, Guangzhou, 510060 China

**Keywords:** MYBL2, Macrophages, Ovarian cancer, CCL2

## Abstract

**Background:**

An immunosuppressive tumor microenvironment in ovarian cancer facilitates tumor progression and resistance to immunotherapy. The function of MYB Proto-Oncogene Like 2 (MYBL2) in the tumor microenvironment remains largely unexplored.

**Methods:**

A syngeneic intraovarian mouse model, flow cytometry analysis, and immunohistochemistry were used to explore the biological function of MYBL2 in tumor progression and immune escape. Molecular and biochemical strategies—namely RNA-sequencing, western blotting, quantitative reverse transcription–polymerase chain reaction (qRT-PCR), enzyme-linked immunosorbent assay, multiplex immunofluorescence, chromatic immunoprecipitation assay (CHIP) and luciferase assay—were used to reveal the mechanisms of MYBL2 in the OVC microenvironment.

**Results:**

We found tumor derived MYBL2 indicated poor prognosis and selectively correlated with tumor associated macrophages (TAMs) in ovarian cancer. Mechanically, C-C motif chemokine ligand 2 (CCL2) transcriptionally activated by MYBL2 induced TAMs recruitment and M2-like polarization in vitro. Using a syngeneic intraovarian mouse model, we identified MYBL2 promoted tumor malignancyand increased tumor-infiltrating immunosuppressive macrophages. Cyclin-dependent kinase 2 (CDK2) was a known upstream kinase to phosphorylate MYBL2 and promote its transcriptional function. The upstream inhibitor of CDK2, CVT-313, reprogrammed the tumor microenvironment and reduced anti-PD-1 resistance.

**Conclusions:**

The MYBL2/CCL2 axis contributing to TAMs recruitment and M2-like polarization is crucial to immune evasion and anti-PD-1 resistance in ovarian cancer, which is a potential target to enhance the efficacy of immunotherapy.

**Supplementary Information:**

The online version contains supplementary material available at 10.1186/s12935-023-03079-2.

## Introduction

Ovarian cancer (OVC) is one of the most common and life-threatening malignancies globally. Although combined surgery and platinum–taxane chemotherapy comprise the first-line treatment for OVC, more than 70% of patients develop platinum resistance and exhibit tumor progression later. Therefore, second-line or alternative therapies are warranted to improve the prognosis of OVC [1].

In the OVC microenvironment, many immunosuppressive cells, such as tumor-associated macrophages (TAMs), regulatory T cells (Tregs), and myeloid-derived suppressor cells (MDSCs), are known to promote malignant tumor progression [2]. Among them, TAMs represent a major infiltrating immune cell subpopulation. Several articles have explained the mechanism of TAM recruitment and polarization in OVC. Adenosine generated by OVC cells likely attracts TAMs infiltration and M2-like polarization by upregulating CD39 and CD73 [3]. Tumor-derived UBR5 promotes TAM recruitment and activation via key cytokines like CCL2 and CSF1 [4]. In recent years, more evidence showed that immunosuppressive M2-like macrophages indicated limited clinical immunotherapy responses [5] and a poor prognosis of OVC patients [6]. These results may bemay be correlated with TAMs causing exhaustion of CD8 + tumor-infiltrating lymphocytes (TILs) [7] or recruitment of other immunosuppressive immune cells [8]. Thus, the investigation of therapeutic targets to block the tumor-promoting functions of TAMs may reveal strategies to enhance the efficacy of OVC treatment.

MYB Proto-Oncogene Like 2 (MYBL2), a *MYB* gene family gene, is broadly expressed in proliferating cells [9]. In past decades, researchers have demonstrated that MYBL2 regulates various biological processes, including cell proliferation, cell differentiation, and apoptosis [10]. MYBL2 is essential to establish definitive hematopoiesis because mice with low levels of MYBL2 developed hematologic disorders during aging [11]. MYBL2 can regulate the cell cycle by interacting with cell cycle regulators and transactivating downstream target genes, such as CCNB1 [12]. The transcriptional function of MYBL2 depends on phosphorylation modification and CDK2 has been identified as a kinase of MYBL2 [13, 14]. MYBL2 also plays a significant role in several types of cancers, such as colorectal cancer [15, 16], prostate cancer [17], and breast cancer [18]. In OVC, MYBL2 deregulated the DREAM complex components [19, 20] and enhanced the malignant progression and olaparib insensitivity of OVC cells by transactivating the cell division cycle associated 8 [21].

A few studies have revealed that MYBL2 is correlated with immune infiltrates in cancer [22, 23]. However, the conclusions of these studies are all drawn by bioinformatic analysis, and the functional involvement and the underlying molecular mechanisms have not been fully elucidated. In the current study, we aimed to explore the undergoing mechanism of MYBL2 causing immune microenvironment reprogramming and anti-PD-1 resistance. The success of this study will deepen the mechanism that MYBL2 induced tumor progression and provide a new treatment strategy for OVC.

## Methods

### Patient populations

129 patients with OVC who underwent primary tumor debulking surgery at Sun Yat-sen University Cancer Center between 2002 and 2014 were enrolled. Written informed consent to participate was obtained from all patients before enrollment. All experimental protocols used in this study were approved by the Ethics Committee of the Sun Yat-sen University Cancer Center Institutional Review Board (approval number: B2022-086-01).

### Cell lines

The human OVC cell lines A2780 and SKOV3, normal ovarian surface epithelial cell line IOSE80, and the mouse OVC cell line ID8 were obtained from Shanghai Bioresource Collection Center (Shanghai, China). All cell lines were cultured in Dulbecco’s modified Eagle’s medium (DMEM) or RPMI-1640 medium supplemented with 10% fetal bovine serum, 100 U/mL penicillin, and 100 mg/mL streptomycin at 37 ℃ in a humidified incubator with 5% CO_2_.

The myeloid cell line THP1 was gifted by Prof. Limin Zheng’s laboratory. THP-1 cells were stimulated with 100 ng/mL phorbol 12-myristate 13-acetate (PMA, Sigma) for 24 h to induce differentiation.

### Cultivation of bone marrow-derived macrophages (BMDMs)

Bone marrow was flushed from the tibias and femurs of C57BL/6 mice, sieved through a 70-µm filter, and red blood cells were lysed using red cell lysis buffer. The remainingcells were resuspended in DMEM plus 10% fetal bovine serum supplemented with 50 ng/ml macrophage colony-stimulating factor (315-02, PeproTech, New Jersey, USA) and cultured at 37 °C in 5% CO2 for 7 days [24]. These BMDMs were used for further experiments.

### Mice and animal experiments

All female C57BL/6 mice (5–6 weeks, female) were purchased from Guangdong Medical Laboratory Animal Center (Guangzhou, China). All mice were handled strictly according to the Principles for the Utilization and Care of Vertebrate Animals and the Guide for the Care and Use of Laboratory Animals. All animal experiments were approved by the Institutional Animal Care and Use Committee of Sun Yat-sen University Cancer Center (21090G).

To establish syngeneic intraovarian models, mice were anesthetized by intraperitoneal injection of 50 mg/kg pentobarbital sodium, and a single dorsal incision was made to access the ovary. ID8 cells (1 × 10^6^) were transduced with virus, then injected into the left ovarian bursa. The tumors were surgically harvested at 8 weeks after ID8 implantation.

For long-term macrophage depletion, mice were intraperitoneally injected with 100 µl (0.05 mg/ml) clodronate liposome (LC) (F70101, FormuMax, California, USA) or phosphate-buffered saline (PBS) liposome on alternative days starting from the 4 fourth weeks after tumor inoculation until 8 weeks.

For antibody treatment, each of the ID8-bearing mice was intraperitoneally administered 250 µg/mouse of anti-PD-1 antibody (BE0273, BioXcell, NewHampshire, USA) at 4 weeks after ID8 inoculation five times on alternate days. For drug treatment, ID8-bearing mice were intraperitoneally administered 20 mg/kg CVT-313 (S6537, Selleck, Texas, USA) for consecutive 12 days, with a break of 1 day every 4 days of treatment at 4 weeks after ID8 inoculation.

### Immunohistochemistry assay

For immunohistochemical analysis, slides were deparaffinized in xylene and alcohol. Antigen retrieval was performed using EDTA (pH 8.0). The tissue sections were blocked in 3% H_2_O_2_ for 10 min and probed with primary antibodies at 4℃ overnight in a moisture chamber. The slides were then rinsed with PBS and incubated with universal immuno-peroxidase polymer anti-Rabbit or anti-Mouse antibodies (#H2008, #2004, NICHIREI BIOSCIENCES Inc. Tokyo, Japan) at 37 ℃ for 0.5 h. The sections were then incubated with a 3,3′-diaminobenzidine tetrahydrochloride substrate (DAB, #K5007, Dako, Denmark) for color development and with hematoxylin for nuclear counterstaining.

Using a tissue microarray (TMA) of OVC samples from the primary cohort, which contained 129 patients, the expression levels of MYBL2 in intratumoral regions and of CD68, CD8, and CD204 in all tissue regions of OVC were determined. To detect and evaluate the MYBL2 signal intensity in the tumor tissue of patients with OVC without bias, stained sections were imaged with the Nuance VIS-FL Multispectral Imaging System and analyzed with the InForm 2.0.1 image analysis software (Perkin-Elmer Applied Biosystems), which enabled tissue compartment (tumor tissue, peritumoral stroma tissue, blank) and cell compartment (cytoplasm, nucleus) segmentation. The DAB object density counts per megapixel for each tissue category were used for further analysis. Immunohistochemical scoring was performed using the histoscore (H-score), which was calculated based on the assessments of both the fraction of positive cells (0–100) and the intensity of staining (0–3) to represent the expression level of MYBL2; the possible scores ranged from 0 to 300. The details are described in our previous article [25]. For immune cell staining, following tissue segmentation, the images were subjected to color deconvolution using the established spectral library, and cell numbers were estimated using the counting object module of InForm 2.0.1 image analysis software. All automated measurements were rechecked by two pathologists who were unaware of the patient’s clinical information. All antibodies were listed in Supplementary Table 1.

For immunohistochemical analysis of mouse tissue, the positively stained cells were counted using Image J software.

### Flow cytometry

Malignant peritoneal wash cells from mice harvested at 8 weeks after ID8 implantation were stained with specific antibodies. For surface staining, cells were collected after erythrocyte segmentation and incubated with Zombie UV Fixable viability dye (423,107, BioLegend, California, USA) for 15 min at room temperature, followed by staining with fluorochrome-conjugated antibodies for 30 min, then washing and analyzing by flow cytometry. The proportion of certain immune subsets was evaluated by a flow cytometer with phenotypic gating criteria. All antibodies were listed in Supplementary Table 1.

### Quantitative reverse transcription–polymerase chain reaction (qRT-PCR) assay

Total RNA was extracted using TRIzol reagent (15,596,026, Thermo Fisher, Massachusetts, USA), and quantified using a Nanodrop 2000 spectrophotometer (Thermo Fisher). Total RNA (2 µg) was converted to cDNA using the GoScript™ Master Mix (A2800, Promega, Wisconsin, USA). The transcripts were quantified by real-time qPCR using a LightCycle 480 instrument (ROCHE 480, ROCHE Diagnostics). The primer sequences are listed in Supplementary Table 2.

### Cell migration assays

Transwell assays were performed to detect THP1 cell migration. Cells were seeded into the upper chamber of a transwell membrane. Conditioned media derived from malignant cells was added into the lower chamber. Cells that migrated to the bottom chamber were fixed with 4% formaldehyde for 20 min and stained with 0.5% crystal violet. Then, five random fields (200× magnification) were observed and photographed using an inverted microscope.

### Immunoblotting assay

For western blotting, cells were collected and lysed in RIPA lysis buffer (#P0013C, Beyotime, Shanghai, China). A bicinchoninic acid assay (#23,227, Thermo Fisher, Massachusetts, USA) was used to measure the protein concentrations in the extracts. Equal amounts of total proteins were separated by SDS-PAGE, and transferred to 0.2 μm polyvinylidenedifluoride membranes (#1,620,177, Bio-Rad, California, USA). The membranes were incubated with the specific antibodies at 4℃ overnight and the signals were measured by ECL reagent (#WBKLS0500, Merck Millipore, New Jersey, USA). All antibodies were listed in Supplementary Table 1.

### The bioinformation analysis

Single-cell sequencing datasets of ovarian cancer were analyzed by TISCH (http://tisch.comp-genomics.org/home/). The correlation between the copy number or expression of *MYBL2* and macrophages was estimated using the TIMER database (https://cistrome.shinyapps.io/timer/, http://timer.cistrome.org/) and The Cancer Genome Atlas (TCGA) database. Differentially expressed genes were subjected to Gene Ontology enrichment analysis using the ClusterProfiler package in R [26]. RNA-sequencing (RNA-seq) raw count data from the TCGA database were estimated using the Tumor Immune Dysfunction and Exclusion (TIDE, https://tide.dfci.harvard.edu/) algorithm to predict the potential ICB response. A low score indicated good efficacy [27].

### Enzyme-linked immunosorbent assay (ELISA)

The concentration of secreted CCL2 in the supernatant collected from culture cells or serum from OVC patients was determined using human ELISA kits according to manufacturers’ instructions (, 438,804, Biolegend, California, USA).

### Immunofluorescence

For the immunofluorescence assay, cells were incubated with primary antibodies overnight at 4℃, followed by incubation with Alexa Fluor 555-conjugated anti-mouse IgG (A32794, Thermo Fisher, Massachusetts, USA) and Alexa Fluor 647-conjugated anti-rabbit antibody (ab150075, Abcam, Cambridge, UK). Confocal imaging was performed using a confocal laser scanning microscope (LSM880, Carl Zeiss, Germany).

### Chromatin immunoprecipitation (CHIP)

CHIP was performed using a CHIP assay kit (SimpleChIPR Plus Sonication Chromatin IP Kit 56,383, Cell Signaling Technology, Massachusetts, USA). Briefly, 5 × 10^6^ SKOV3 cells were fixed with formaldehyde, quenched with glycine at room temperature, and then collected, washed, and resuspended in a lysis buffer. Sonicated chromatin solution was immunoprecipitated with anti-IgG and anti-MYBL2 antibodies (A301-656 A-T, ThermoFisher, Massachusetts, USA). Immunoprecipitated DNA was purified by column collection and analyzed by qPCR.

### Luciferase reporter assay

A2780 and SKOV3 cells were transfected with the indicated plasmids (Supplemental methods). The firefly and renilla luciferase activities were measured using the Dual-Luciferase Kit (RG028, Beyotime, Shanghai, China) according to the manufacturer’s instructions.

### Statistical analysis

Overall survival was defined as the interval from surgery to the date of death or the date of the last follow-up visit. Kaplan–Meier analysis was used to estimate the survival outcomes. All statistical analyses were performed using SPSS 22.0 (Chicago, IL, USA). Experimental data were analyzed using an independent samples t-test to compare two groups and a one-way ANOVA analysis of variance to compare multiple groups. Spearman’s rank correlation test was used to determine statistical correlations. Statistical significance was set at *P* < 0.05.

## Results

### Tumor-derived MYBL2 is correlated with tumor progression and macrophage infiltration in OVC

**Fig. 1:**
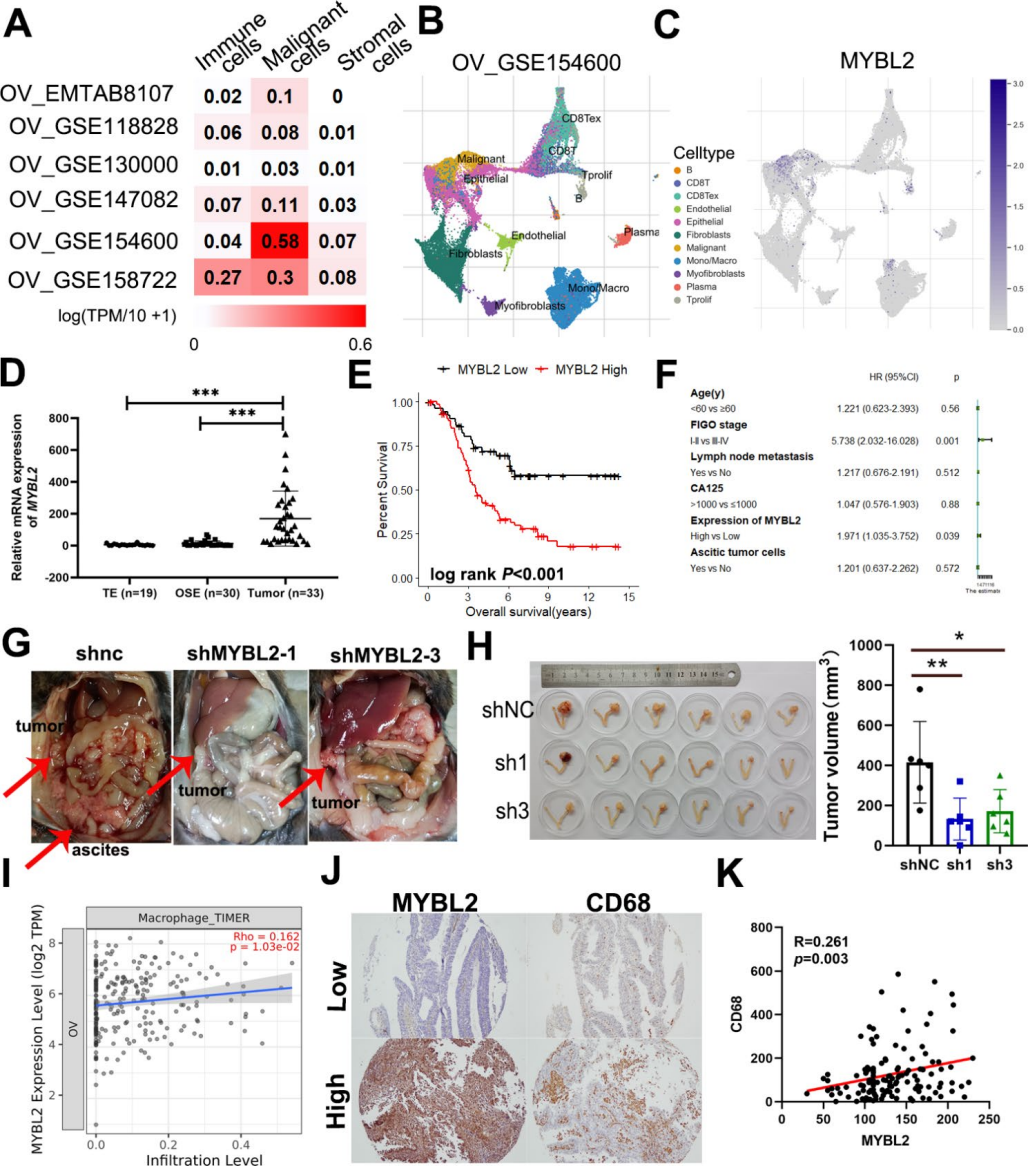
Tumor derived MYBL2 promotes tumor malignancy and macrophage abundance. (**A**) The expression of MYBL2 was analyzed from single cell sequencing datasets of ovarian cancer by TISCH. (**B-C**) The representative dataset of ovarian cancer and the MYBL2 expression in all cell types. (**D**) The *MYBL2* mRNA expression in human ovarian surface epitheliums and ovarian tumors. (**E**) Overall survival (OS) curves based on MYBL2 expression were obtained using the Kaplan–Meier method and analyzed using the log-rank test. (**F**) Forest plot showing the results of a multivariate analysis of factors associated with OS. Ascites (**G**), and tumors (**H**) from orthotopic syngenetic mouse models. (**I**) Association between the abundance of macrophages and mRNA expression of MYBL2 in OVC. (**J-K**) Association between the expression of MYBL2 and the expression of CD68 by immunohistochemistry

Gene expression microarrays showed that *MYBL2* expression was elevated in OVC tissues compared to normal ovarian tissues (Supplemental Fig. 1A-B). We found MYBL2 expression level remains the highest in malignant OVC cells from 6 single-cell datasets of the TISCH database (Fig. 1A). Especially in OV_GSE154600, malignant cells showed dominant expression of MYBL2 (Fig. 1B-C). We used qPCR to further confirm the MYBL2 expression in OVC tissue (Fig. 1D), which is consistent with the gene expression data from the western population (TCGA/GTEx/GSE10971; Supplemental Fig. 1C-D). To determine the clinical value of *MYBL2* in OVC, we found an association between elevated *MYBL2* expression and reduced survival by online Kaplan–Meier plotter data set analysis (Supplementary Fig. 1E). In addition, immunohistochemical staining for MYBL2 in 129 OVC tissues demonstrated a similar trend of correlation between high MYBL2 expression and worse prognosis (Fig. 1E). Multivariate analyses revealed that the FIGO stage (*P* = 0.001) and MYBL2 expression (*P* = 0.039) were independent prognostic factors (Fig. 1F).

To investigate the functional importance of MYBL2 in OVC development, we generated a mouse model of ovarian cancer by injection of syngenetic ID-8 mouse ovarian cancer cells with lentivirus encoding green fluorescent protein (GFP)-tagged control scrambled shRNA (shNC) or MYBL2-targeting shMYBL2 (shMYBL2-1, shMYBL2-3) into the right ovarian bursae of C57BL/6 mice (Supplementary Fig. 1F). We found that the shNC group developed more obvious hemorrhagic ascites (Fig. 1G, Supplementary Figure G-H), and compared with the shNC group, the solid tumors in the shMYBL2 group were significantly smaller in immunocompetent mice (Fig. 1H). We also found that tumor-induced splenomegaly was more apparent in the shNC group than the shMYBL2 group (Supplementary Figure I). Extensive dissemination metastasis in the pelvis cavity as well as the abdominal cavity, and hematogenous dissemination of lung metastases were far more tumor.

masses in ID8-bearing shNC-treated mice than in shMYBL2-treated mice (Supplementary Figure J-K).

To further investigate the association between MYBL2 and lymphocyte infiltration, we analyzed TCGA data and found that *MYBL2* expression was slightly positively associated with the abundance of macrophages (Fig. 1I). Given the cluster of differentiation 68 (CD68) is highly expressed in the monocyte lineage, circulating macrophage, and tissue-resident macrophages [28]. We analyzed the correlation by performing immunohistochemical staining for CD68 in serial tumor tissue slices from the SYSUCC cohort patients. The findings confirmed that high MYBL2 expression was positively correlated with the number of CD68 + cells (Fig. 1J-K). These results suggest that high MYBL2 expression may be associated with more macrophages infiltration.

### MYBL2 transcriptionally induced CCL2 expression in OVC

**Fig. 2:**
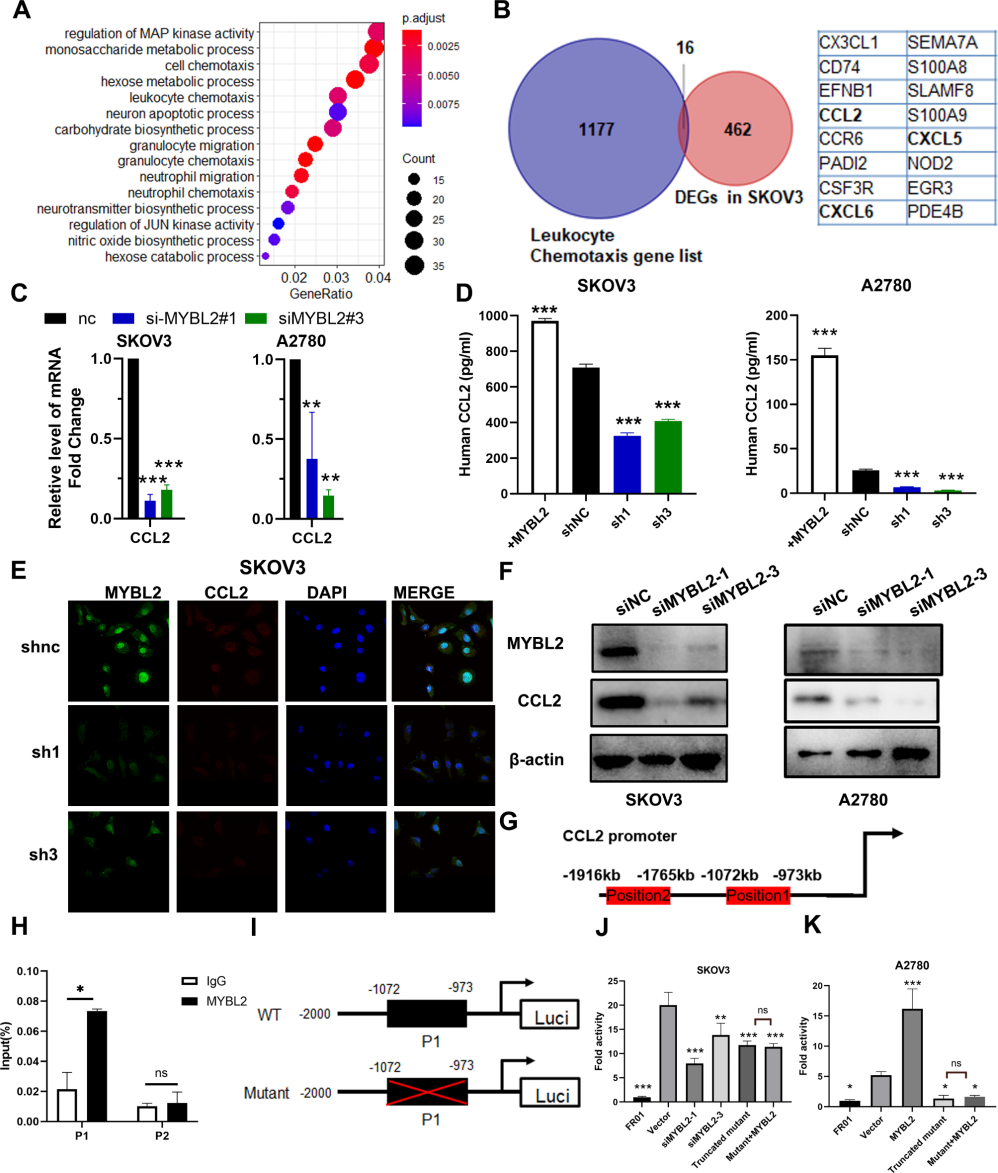
MYBL2 transcriptionally activates CCL2 expression. (**A**) Gene Ontology term analyses of significantly differentially expressed genes in the shMYBL2 group compared with the vector control group. (**B**) Venn diagram of genes involved in the leukocyte chemotaxis pathway and significantly differentially expressed genes. Quantitative PCR (**C**), ELISA (**D**), immunofluorescence (**E**), and western blotting (**F**) were used to detect CCL2 expression. (**G-H**) CHIP assay to identify the possible binding site of MYBL2 on the CCL2 promoter. (**I-K**) Relative luciferase activities of different reporters containing wild-type or mutant sequences of the CCL2 promoter in the indicated cells

To explore the mechanism underlying the association between MYBL2 and TAMs infiltration, we performed RNA-seq of SKOV3/shNC and SKOV3/shMYBL2-1 cells and found that 462 genes were differentially expressed following MYBL2 knockdown (false discovery rate < 0.05, Log2 > 1.5). Further bioinformatic analysis revealed that pathways related to immunomodulation, including the leukocyte chemotaxis pathway, were significantly enriched upon MYBL2 knockdown (Fig. 2A). We next identify 16 intersecting genes between the leukocyte chemotaxis pathway and differentially expressed genes in SKOV3 cells (Fig. 2B). Among them, CCL2, CXCL5 and CXCL6 encoded chemokines. The fold change for CCL2, CXCL5, and CXCL6 was − 1.75, -3.16, and − 3.26, respectively. Studies have shown that CCL2 is crucial in mediating monocyte/macrophage chemotaxis and functional suppression (Zhang et al., 1994). However, CXCL5 and CXCL6 are regarded as cytokines that mainly attract neutrophilic granulocytes (Linge et al., 2008; Wuyts et al., 1999). Thus, we speculated that CCL2 may be a key downstream effector molecule of MYBL2. We validated the results of RNA-seq by qRT-PCR and found that CCL2 expression was significantly changed (Fig. 2C). With the overexpression of MYBL2 in IOSE80 cells, the expression of CCL2 also increased (Supplementary Fig. 2A). We evaluated the concentrations of cytokines in two OVC cell lines by ELISA. CCL2 expression was much higher in the supernatants of shNC cells than in the conditioned medium of shMYBL2 cells. Furthermore, CCL2 expression was upregulated in the supernatants of MYBL2-overexpressing cells (Fig. 2D). Consistently, MYBL2 knockdown downregulated CCL2 expression, as confirmed by laser-induced fluorescence confocal microscopy (Fig. 2E, Supplementary Fig. 2B) and western blotting (Fig. 2F).

As a transcription factor, MYBL2 regulates the expression of downstream genes by binding to their promoters. The potential binding region of the CCL2 promoter was predicted by JASPAR (Fig. 2G). Then, we performed a CHIP assay to examine whether MYBL2 is bound to the CCL2 promoter region. The findings revealed that the position 1 which indicated the CCL2 promoter region between − 1072 to -973 bp showed MYBL2 enrichment (Fig. 2H). A luciferase reporter assay showed that MYBL2 was capable of enhancing CCL2 promoter activity. MYBL2 downregulation reduced fluorescence intensity ratio change in SKOV3 cells, whereas MYBL2 overexpression upregulated it in A2780 cells, which was consistent with the *CCL2* mRNA expression level. And the mutation of the CCL2 promoter region between − 1072 to -973 bp dramatically abolished the promoter’s responsiveness to MYBL2 in A2780 and SKOV3 cells. MYBL2 overexpression in the context of a mutated CCL2 promoter region between − 1072 to -973 bp showed no effect on luciferase activity (Fig. 2I-K).

### MYBL2-CCL2 axis modulated macrophage infiltration and M2-like macrophage polarization

**Fig. 3:**
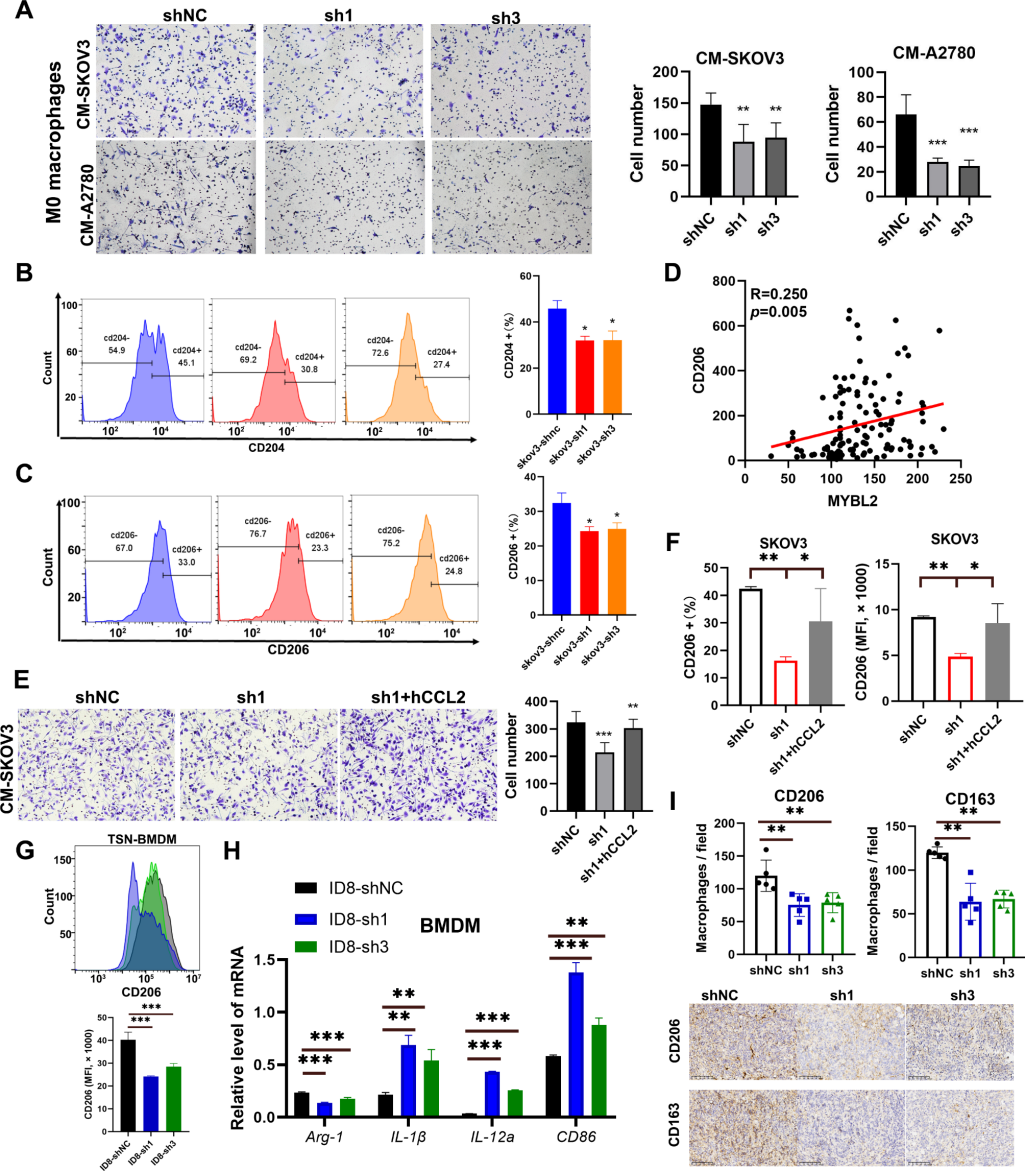
MYBL2-CCL2 axis promoted recruitment and M2 polarization of macrophages in vitro. (**A**) Chemotactic migration assays of macrophages using the supernatant of SKOV3 or A2780 cells. (**B-C**) Flow cytometry analysis of CD204 + cells and CD206 + cells in PMA-stimulated THP-1 cells treated with the supernatant of SKOV3 and A2780 cells for 48 h. (**D**) Association between the expression of MYBL2 and the expression of CD206 by immunohistochemistry. (**E**) Chemotactic migration assays of macrophages using the supernatant of SKOV3 cells. (**F**) Flow cytometry analysis of CD206 + proportion and expression in PMA-stimulated THP-1 cells treated with the supernatant of SKOV3 cells for 48 h. (**G**) Flow cytometry analysis of CD206 expression in bone marrow-derived macrophages (BMDMs) treated with the supernatant of ID8 cells for 24 h. (**H**) Quantitative PCR analysis of Arg-1, IL-1β, IL-12a, and CD86 in BMDMs treated with the supernatant of ID8 cells for 24 h. (**I**) CD206 and CD163 immunohistochemical staining and quantification in the tumors of mice

To clarify the effect of the MYBL2-CCL2 axis on macrophages, we treated THP1 cells with PMA to induce differentiation into M0 macrophages and performed a transwell assay to analyze the migration of M0 macrophages from different conditioned media (CM)in the lower chamber. As shown in Fig. 3A, the number of migrated cells from shMYBL2-1 and shMYBL2-3 CM were significantly lower, indicating that MYBL2 inhibition reduced macrophage migration.

Both the number of TAMs numbers and the phenotype of macrophages have important implications for cancer progression and clinical outcomes. To further evaluate the functional changes of macrophages, we treated M0 macrophages with different conditioned medium from SKOV3 treated with shNC or shMYBL2, and then analyzed the percent of CD204 + and CD206 + cells by flow cytometry. CD204 and CD206 were general markers indicating M2-like macrophage polarization. We found that macrophages treated with CMderived from shMYBL2 cells exhibited fewer CD204 + cells and CD206 + cells by flow cytometry analysis (Fig. 3B-C). The macrophages treated with CMderived from A2780-shMYBL2 cells also showed a lower proportion of CD204 + cells (Supplementary Fig. 3A). The results of immunohistochemical staining in OVC patients also showed that MYBL2 expression was positively correlated with the number of CD206 + and CD204 + cells (Fig. 3D, Supplementary Fig. 3B). The CM derived from shNC cells also suppressed levels of M1-like markers: *IL-1β*, *IL-12 A*, and *CD86* by qPCR (Supplementary Fig. 3C-D).

Next, we investigated whether CCL2 is a primary effector of MYBL2-mediated processes. We identified that the migration effect reduced by shMYBL2 was almost restored by added CCL2 (1000 pg/ml) (Fig. 3E). CM derived from MYBL2-knockdown cells attenuated CD206 expression and proportion in M0 macrophage cells compared with control cells, and this effect was rescued by CM with added h-CCL2 (Fig. 3F, Supplementary Fig. 3E).

We also verified these results in BMDMs, and found that the conditioned medium derived from shMYBL2 cells reduced CD206 expression compared with cells treated with supernatants of shNC cells (Fig. 3G). The mRNA expression levels of genes characteristics of a tumor-supportive macrophage phenotype, including *Arg-1*, were also downregulated by shMYBL2-CM, while those of the cytotoxic response-related genes *IL-1β*, *IL-12 A*, and *CD86* were increased by supernatants of shMYBL2 cells (Fig. 3H). To further investigate the polarization of TAMs, we assessed CD206 and CD163 expression to detect M2-like TAMs in solid tumors and found that M2-like TAMs were significantly reduced in shMYBL2-1 and shMYBL2-3 groups (Fig. 3I). Together, these results indicate that MYBL2 promoted macrophage recruitment and M2-like polarization largely through CCL2.

### MYBL2 knockdown reduces TAMs in ovarian masses and ascites

**Fig. 4:**
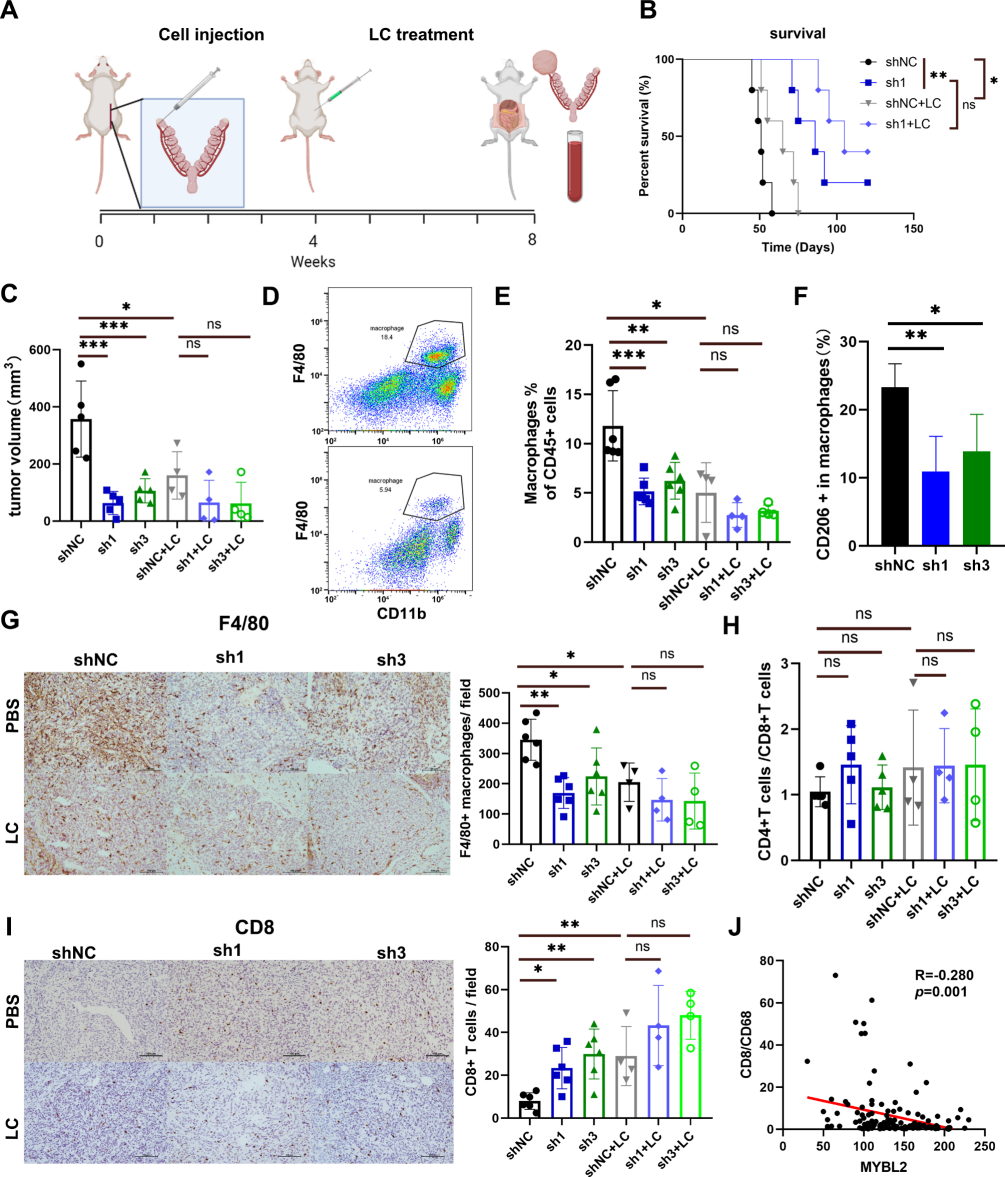
Impaired macrophage recruitment and tumor growth in vivo upon shMYBL2 of mice with OVC. (**A**) Workflow of investigation with or without clodronate liposome (LC) treatment. (**B**) Overall survival (OS) curves were obtained using the Kaplan–Meier method and analyzed using the log-rank test. (**C**) Tumors from orthotopic syngenetic models with or without LC treatment. Flow cytometry analysis of the proportions of infiltrated CD11b + F4/80 + macrophages (**D-E**), CD206 + cells in macrophages (**F**) and the ratio of CD4+/CD8 + T cells in ascites (**H**). F4/80 (**G**) and CD8 (**I**) immunohistochemical staining and quantification in the tumors of Lipo-PBS and Lipo-clodronate-treated mice. (**J**) Association between the expression of MYBL2 and the ratio of CD8/CD68 by immunohistochemistry

To further explore whether MYBL2 knockdown influence the TAMs, we depleted macrophages in ID8-bearing mice with liposome-encapsulated clodronate (LC) (Fig. 4A). Survival analysis revealed that mice benefited from the LC treatment and MYBL2 knockdown (Fig. 4B). We measured the tumor volume to determine whether impaired tumor growth upon MYBL2 knockdown was a consequence of reduced macrophage recruitment. We noticed that LC treatment obviously reduced tumor growth. In addition, the ovarian masses in the ID8-bearing GFP-treated group were significantly larger than those in the ID8-bearing shMYBL2-treated group, but the difference was eliminated by the LC treatment (Fig. 4C, Supplementary Fig. 4A-B). This finding indicates that macrophages promoted tumor progression and the effect of shMYBL2 on tumor was dependent on the immunocompetent microenvironment, especially TAMs. We performed flow cytometry analysis of cells in ascites to investigate the proportions of macrophages and T cells (Supplementary Fig. 4C). The flow cytometry results showed that TAMs (F4/80 + CD11b + cells) in ascites were significantly reduced in shMYBL2 groups. LC treatment depleted TAMs successfully and lead to the proportion of TAMs reduced to lower than 5% (Fig. 4D-E). The CD206 + macrophages were also fewer in the shMYBL2 groups than the shNC group (Fig. 4F). Immunohistochemical staining of solid tumor revealed consistent results (Fig. 4G). GFP + tumor cells in ascites were also significantly reduced in shMYBL2 groups (Supplementary Fig. 4D). T cells are regarded as the most important executors of adaptive anti-tumor immunity. Thus, we also performed a flow cytometry analysis of T cells in ascites. The CD4 + T /CD8 + T cell ratio was almost the same between the shMYBL2 and shNC groups (Fig. 4H). However, CD8 + T cells in solid tumor were significantly increased in the shMYBL2 groups, the difference disappeared upon LC treatment, which suggested the upregulation of CD8 + T cells might be an adaptive change after macrophage depletion (Fig. 4I). In OVC tumor tissues, although MYBL2 expression was not associated with the number of CD8 + cells (Supplementary Fig. 4E), it was positively correlated with the CD68+ /CD8 + cells ratio (Fig. 4J). These results confirm that MYBL2 promoted macrophages recruitment and M2-like polarization in vivo.

### Inhibition of MYBL2 improves the efficacy of anti-PD-1 therapy

**Fig. 5:**
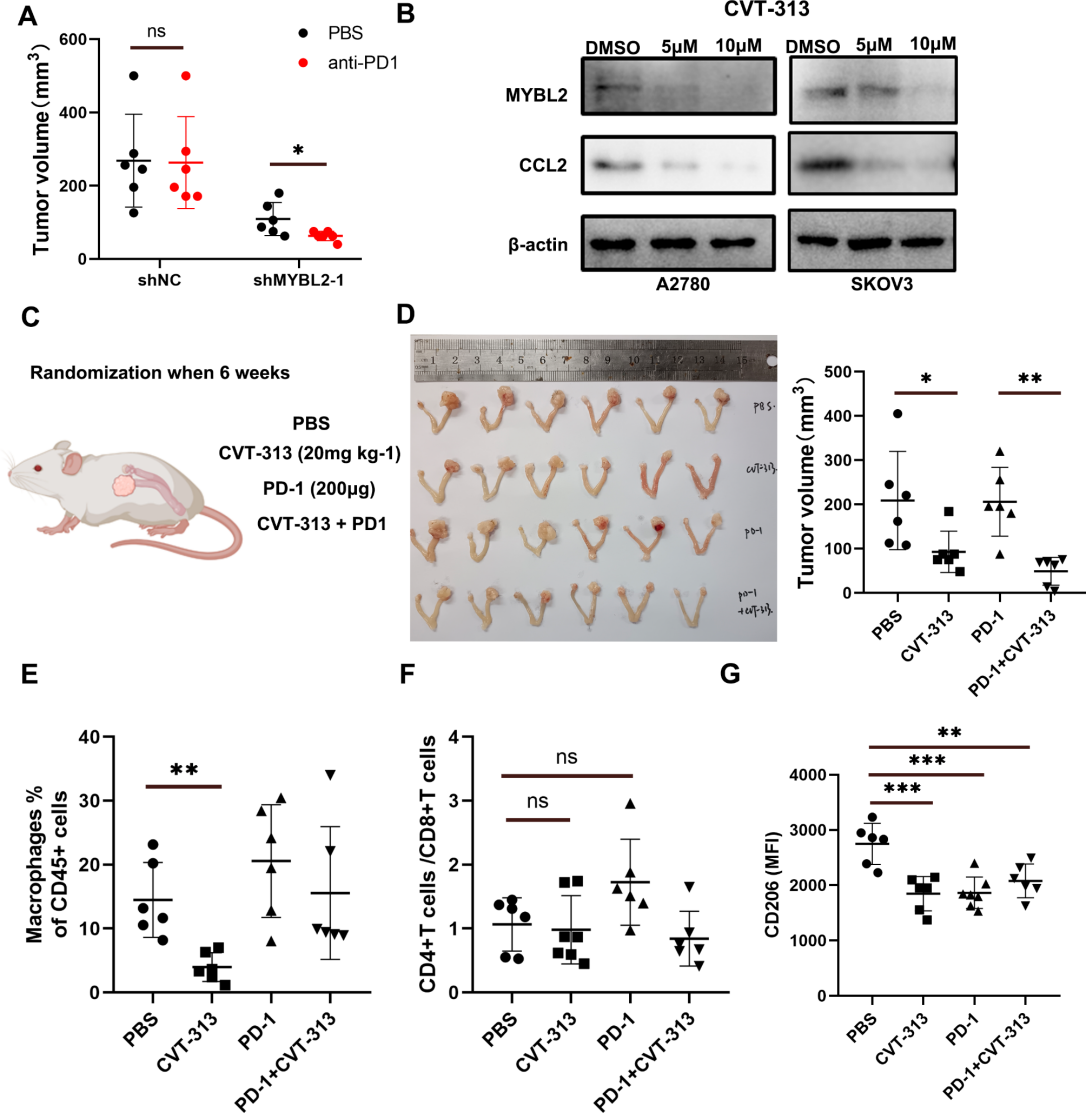
Inhibition of MYBL2 reduces resistance to anti-PD1 therapy in ovarian cancer. (**A**) MYBL2 knockdown reduces anti-PD1 resistance in orthotopic syngenetic model mice. (**B**) CVT-313 shows different effects on the MYBL2–CCL2 axis. (**C**) ID8 cells were injected into the left ovarian bursa. After 6 weeks, mice were treated with anti-PD1, CVT-313, or combination therapy. (**D**) Tumor volumes in the orthotopic syngenetic model mice. (**E-G**) The proportion of infiltrated CD11b + F4/80 + macrophages, expression of CD206 in macrophages, and ratio of CD4+/CD8 + T cells in ascites

The TIDE algorithm was used to predict the therapeutic effect of ICB therapy. The results revealed that in OVC, lower *MYBL2* expression was associated with a lower TIDE score, including that these patients may benefit from ICB therapy (Supplementary Fig. 5A). Analyses of the Gene Expression Omnibus public sequencing data indicated a lower level of *MYBL2* mRNA in anti-PD-1 responders than in progressors with melanoma (GSE78220) (Supplemtary Fig. 5B). We analyzed the expression of PD-1 and PD-L1 of all immune cells in mice and found these two markers had a trend of higher expression in shMYBL2 groups without statistical significance (Supplementary Fig. 5C). Using syngeneic intraovarian mouse models, we showed that anti-PD-1 therapy had little effect on tumor growth in the ID8-bearing shnc group. However, the group with MYBL2 inhibition that received anti-PD-1 therapy showed significantly reduced tumor volume compared to the group without anti-PD-1 therapy (Fig. 5A).

No direct MYBL2 Inhibitors are currently available; however, it has been reported that targeting its major upstream cyclin-dependent kinase (CDK), which respectively regulates MYBL2 expression through phosphorylation, is a potential new therapeutic approach to inhibit MYBL2 expression and its downstream target gene expressions [29]. Thus, we treated OVC cells with CTV313, which targeted CDK2. We found that CVT313 strongly reduced MYBL2 and CCL2 expression, as revealed by western blotting (Fig. 5B) and slightly reduced cell viability (Supplementary Fig. 5D). Using orthotopic xenograft tumor mouse models (Fig. 5C), we showed that the tumor volume and the abundance of macrophages in ascites were significantly lower in the CVT-313-treated group than in the PBS-treated control group (Fig. 5D-E, supplementary Fig. 5E). Importantly, we found that the mean fluorescence intensity of CD206 expression was also significantly reduced in the CVT-313, anti-PD-1, and combination therapy groups compared with the PBS-treated control group, but the CD4+/CD8 + cell ratio was not different between these groups (Fig. 5F-G).

## Discussion

This study showed that tumor-derived MYBL2 transcriptionally activated *CCL2* and recruited M2-like macrophages within clinical tumor tissues and model mice, and thus structured the immunosuppressive OVC microenvironment and attenuated the response to anti-PD-1 therapy. This finding reveals a previously unidentified mechanism of macrophage infiltration in OVC.

Although more studies strongly proposed that ovarian cancer originated from the fallopian tube [30, 31], the intra-ovarian model of syngeneic or orthotopic high-grade OVC model surgically implanted into the ovary in mice was commonly used in ovarian cancer microenvironment research [4, 32, 33]. The mouse model developed early serous carcinomas in the fallopian tube had limited application in fundamental research [34].

MYBL2, a key transcriptional factor associated with the cell cycle, plays a pivotal role in cancer progression. In OVC, patients with high MYBL2 expression have been showed to exhibit downregulated DREAM-mediated cell cycle gene expression and poor survival [19, 20]. Several studies have investigated the relationship between MYBL2 expression and an immunosuppressive tumor microenvironment, using online or offline bioinformatic tools in pan-cancer analyses and have proposed that MYBL2 can affect the tumor immune microenvironment by influencing the immune infiltration levels and expression levels of CD4 + T cells, CD8 + T cells, cancer-associated fibroblasts, and immune checkpoint-associated cells [22]. However, few studies have examined the specific functional and clinical implications of MYBL2 in the OVC immune microenvironment. We demonstrated that MYBL2 expression was positively associated with the abundance of TAMs in both clinical OVC tissue and online database. However, our syngeneic intraovarian mouse models demonstrated that MYBL2 failed to induce intratumoral and ascitic trafficking of TAMs and facilitated tumor progression upon LC treatment, indicating that the tumor-promoting mechanisms of MYBL2 largely depended on TAMs. In the solid tumors of mice, MYBL2 knockdown significantly increased the abundance of CD8 + T cells, but this change disappeared upon LC treatment. Interestingly, MYBL2 expression showed no relationship with CD8 + T cells but had a negative association with the CD8+/CD68 + cell ratio in OVC clinical tissue. Accordingly, the increased abundance of CD8 + T cells in mice was the attendant result after weakened TAMs recruitment. As for the reason why MYBL2 expression appeared to have no relationship with CD8 + T cells in clinical tissue, we hypothesize that tumor progression in patients involves a complex regulation network and other pathways may affect the abundance of CD8 + T cells in the microenvironment with low MYBL2 expression.

Our results of RNA-seq data analysis suggest that MYBL2 knockdown downregulates immune-associated pathway components, including several cytokines and chemokines; therefore, we investigated CCL2 expression downstream of MYBL2, which functions as a chemokine to attract TAMs into tumors. Recent studies have reported on the CCL2–CCR2–M2 macrophage axis in OVC. One study showed that CCR2 blockade by a CCR2 antagonist in vivo decreased the number of M2 macrophages, increased the number of IFNγ + CD8 + T cells, and inhibited tumor growth, indicating that M2 macrophage-mediated indirect effects significantly affect tumor growth [35]. Few articles explored the association between tumor-derived CXCL5, CXCL6 and TAMs.

CCL2 is a key regulator of TAM recruitment, differentiation and function and is produced by both tumor cells and non-tumor cells [36]. Because CCL2 production in tumors facilitates the accumulation of immune-suppressive, tumor-promoting TAMs, it has become a molecular target for cancer treatment [37]. In this study, MYBL2 transcriptionally activated *CCL2* and further promoted the recruitment and M2-like polarization of macrophages, thereby inducing tumor progression and anti-PD-1 therapy tolerance. Thus, blocking the MYBL2–CCL2 axis might be a promising treatment strategy to control tumor progression and enhance sensitivity to anti-PD-1 therapy. Although anti-CCL2 antibodies and CCR2 antagonists have been tested in clinical trials, one study reported that the interruption of anti-CCL2 antibody treatment markedly increased lung metastasis and accelerated death in mouse syngeneic cancer models [38]. Thus, the blockade of the CCL2–CCR2 pathway alone remains controversial [39].

Multiple regulatory networks contribute to ICB resistance. OVC is known to respond poorly to ICB with clinical trials reporting responses ranging from 6–22% [40, 41]. Therefore, to improve the OVC response rate to ICB inhibitors in clinical practice, combinations of ICB with chemotherapy, targeted therapy, and radiation have been proposed [42–44]. Among the combination therapies, targeting aberrant oncogenic signaling pathways, including the MAPK, PI3K–AKT–mTOR, and JAK–STAT pathways, in cancer cells has been shown to increase the efficacy of ICB in preclinical and clinical studies [45]. Blocking the genes upstream of the MYBL2 oncogene instead of the CCL2–CCR2–macrophage axis might be another approach to improve the ICB efficacy. As no direct MYBL2 inhibitor is currently available, we chose the inhibitor of its major upstream gene *CDK2*. Although several CDK2 inhibitors have been clinically evaluated, most of them also target other CDKs [46]. Selective CDK2 inhibitors have not been widely used in tumor research. CVT-313 is a potent and selective CDK2 inhibitor with significant effects on MYBL2 and CCL2 expression [47]. In our vivo experiment, CVT-313 significantly reduced the tumor volume and enhanced the tumor sensitivity to anti-PD-1 therapy, indicating its potential application in clinical practice.

Not all ICB therapy inhibitors have shown curative effects in mice models of OVC [48, 49]. As for the immune microenvironment change in the ICB therapy alone group, we found that the macrophages and the CD4 + T cells /CD8 + T cells ratio in ascites showed was significantly upregulated after anti-PD-1 therapy. It will be of great interest to explore how this regulation is affected in our future research.

This study has several limitations. As we found no relationship between MYBL2 and CD8 expression in OVC clinical tissue, we did not explore the direct immunomodulatory effect of MYBL2 on T cells. Although several studies have reported that M2 macrophages suppress T cell functions [50], we did not identify immunosuppressive effects on T cell activity indirectly mediated through M2 TAMs. We found CXCL5 was also significantly reduced upon MYBL2 knockdown. CXCL5 is one of the main chemokines that attract MDSCs, which are important regulators of immune responses, to the tumor [51]. However, we did not investigate whether MYBL2 recruited MDSCs and thus rebuilt the immune microenvironment. Finally, the relationships between MYBL2 expression and other biomarkers of ICB, such as the tumor mutation burden, remain unknown.

## Conclusions

We demonstrated that the MYBL2–CCL2 axis favors a tumor inhibitory effect by promoting the M2-like polarization of recruited macrophages, and that CVT-313 reshapes the tumor microenvironment and reverses resistance to anti-PD-1 therapy. Our study provides a promising therapeutic regimen involving combination therapy with anti-PD-1 for OVC.

### Electronic supplementary material

Below is the link to the electronic supplementary material.

Supplementary Material 1

## Data Availability

Please contact corresponding author for data requests.

## Abbreviations

OVC: Ovarian cancer
MYBL2: MYB Proto-Oncogene Like 2
CCL2: C-C Motif Chemokine Ligand 2
TME: Tumor microenvironment
TAMs: Tumor-associated macrophages
Tregs: Regulatory T cells
MDSCs: Myeloid-derived suppressor cells
BMDMs: Bone marrow-derived macrophages
GTEx: The Genotype-Tissue Expression project
TCGA: The Cancer Genome Atlas
CHIP: chromatin immunoprecipitation
RNA-seq: RNA sequencing
qRT-PCR: Quantitative reverse transcription–polymerase chain reaction
GO: Gene Ontology
ICB: Immune checkpoint blockade
CM: Conditioned media
