# Supplementary material for: The MYBL2–CCL2 axis promotes tumor progression and resistance to anti-PD-1 therapy in ovarian cancer by inducing immunosuppressive macrophages
Source: Cancer Cell Int. 2023 Oct 21;23:248. doi: 10.1186/s12935-023-03079-2 (PMC10590509; doi:10.1186/s12935-023-03079-2)

**Supplementary Methods**

**Cell proliferation assays**

The cells were seeded in 96-well plates at 2000 cells per well for the cell proliferation assays. Cell proliferation was evaluated by 10% CCK8 (B34302, bimake, USA) diluted in standard culture media for 2h. Proliferation rates were determined at 0, 24, 48, 72, 96 h after seeding.

**Small interfering RNA (siRNA), short hairpin RNA (shRNA), and plasmid constructs**

All siRNAs were designed and synthesized by JiJie (Guangzhou, Guangdong, China) all the siRNA sequences are listed in Supplementary Table 3. MYBL2 shRNAs were designed based on siRNA sequences. Plasmids targeting MYBL2 shRNA transfected into HEK-293T with pLP1, pLP2 and pLP/VSVG to product virus. The OVC cells were injected with viral supernatants and selected by puromycin (2μg/ml) to establish the stable cell lines. The luciferase reporter plasmids were designed and synthesized by FuNeng (Shanghai, China).

**Supplementary Table 1**

| **Antibody** | **Species Reactivity** | **Application** | **Dilution** | **Supplier** | **Catalog number** |
| --- | --- | --- | --- | --- | --- |
| MYBL2 | Human | IHC | 1:500 | ThermoFisher, USA | PA5-46845 |
| CD8 | Human | IHC | 1:100 | ZSBio, China | ZA-0508 |
| CD68 | Human | IHC | 1:200 | ZSBio, China | ZM-0060 |
| CD204 | Human | IHC | 1:100 | ThermoFisher, USA | 14-9054-80 |
| CD206 | Human | IHC | 1:200 | R&D Systems | MAB25341 |
| CD8a | Mouse | IHC | 1:1000 | Cell Signaling  Technology, USA | 98941T |
| F4/80 | Mouse | IHC | 1:1000 | Cell Signaling  Technology, USA | 98941T |
| CD206 | Mouse | IHC | 1:1000 | Cell Signaling  Technology, USA | 24595T |
| CD163 | Mouse | IHC | 1:1000 | Abcam, UK | ab182422 |
| PD-1 | Mouse | IHC | 1:50 | Bioxcell, USA | BE0273 |
| PD-L1 | Mouse | IHC | 1:200 | Cell Signaling  Technology, USA | 84651T |
| CD45-AF700 | Mouse | FC |  | Biolegend, USA | 103127 |
| CD3-AF700 | Mouse | FC |  | Biolegend, USA | 100215 |
| CD4-PE | Mouse | FC |  | Biolegend, USA | 100511 |
| CD8a-APC | Mouse | FC |  | Biolegend, USA | 100711 |
| CD11b-APC | Mouse | FC |  | Biolegend, USA | 101211 |
| F4/80-PE | Mouse | FC |  | Biolegend, USA | 123109 |
| CD206-PE/Cy7 | Mouse | FC |  | Biolegend, USA | 141719 |
| CD206-PC7 | Human | FC |  | Biolegend, USA | 321124 |
| CD206-FITC | Human | FC |  | Biolegend, USA | 321103 |
| CD204-APC | Human | FC |  | Biolegend, USA | 371905 |
| PD-L1-PE | Human | FC |  | Biolegend, USA | 329706 |
| MYBL2 | Human | WB  IF | 1:300  1:100 | Proteintech, China | 188916 |
| CCL2 | Human | WB | 1:1000 | Affinity Biosciences, China | BF0556 |
| β-actin | Human | WB | 1:1000 | Cell Signaling  Technology, USA | 8457 |
| NF-κB | Human | WB | 1:1000 | Cell Signaling  Technology, USA | 8242 |
| p-NF-κB | Human | WB | 1:1000 | Cell Signaling  Technology, USA | 3033 |
| CREB | Human | WB | 1:1000 | Cell Signaling  Technology, USA | 9197 |
| p-CREB | Human | WB | 1:1000 | Cell Signaling  Technology, USA | 9198 |
| CCL2 | Human | IF | 1:200 | Affinity Biosciences, China | BF0556 |
| MYBL2 | Human | IP | 1:50 | ThermoFisher, USA | A301-656A |

**Supplementary Table 2**

| **Primer** | | **Sequences (5’-3’)** |
| --- | --- | --- |
| **For RT-qPCR** | | |
| MYBL2 | Forward | CCGGAGCAGAGGGATAGCA |
|  | Reverse | CAGTGCGGTTAGGGAAGTGG |
| β-actin | Forward | CACCATTGGCAATGAGCGGTTC |
|  | Reverse | AGGTCTTTGCGGATGTCCACGT |
| CCL2 | Forward | CAGCCAGATGCAATCAATGCC |
|  | Reverse | TGGAATCCTGAACCCACTTCT |
| CCL20 | Forward | AAGTTGTCTGTGTGCGCAAATCC |
|  | Reverse | CCATTCCAGAAAAGCCACAGTTTT |
| CXCL5 | Forward | CAGACCACGCAAGGAGTTCATC |
|  | Reverse | TTCCTTCCCGTTCTTCAGGGAG |
| CXCL8 | Forward | GAGAGTGATTGAGAGTGGACCAC |
|  | Reverse | CACAACCCTCTGCACCCAGTTT |
| IL-1β | Forward | CCACAGACCTTCCAGGAGAATG |
|  | Reverse | GTGCAGTTCAGTGATCGTACAGG |
| IL-12a | Forward | TGCCTTCACCACTCCCAAAACC |
|  | Reverse | CAATCTCTTCAGAAGTGCAAGGG |
| CD86 | Forward | CCATCAGCTTGTCTGTTTCATTCC |
|  | Reverse | GCTGTAATCCAAGGAATGTGGTC |
| mArg-1 | Forward | CATTGGCTTGCGAGACGTAGAC |
|  | Reverse | GCTGAAGGTCTCTTCCATCACC |
| mIL-1β | Forward | TGGACCTTCCAGGATGAGGACA |
|  | Reverse | GTTCATCTCGGAGCCTGTAGTG |
| mIL-12a | Forward | ACGAGAGTTGCCTGGCTACTAG |
|  | Reverse | CCTCATAGATGCTACCAAGGCAC |
| mCD86 | Forward | ACGTATTGGAAGGAGATTACAGCT |
|  | Reverse | TCTGTCAGCGTTACTATCCCGC |
| mβ-actin | Forward | CATTGCTGACAGGATGCAGAAGG |
|  | Reverse | TGCTGGAAGGTGGACAGTGAGG |
| **For ChIP-qPCR** | | |
| CCL2-P1 | Forward | CACTGCTGCCTGCTATGCTA |
|  | Reverse | ACTAGTTGCCTGCTTGGGTC |
| CCL2-P2 | Forward | TTGAGGCATGAGCAGAGGACT |
|  | Reverse | GCCATTTCAGACTCTGGTGGA |

**Supplementary Table 3**

| **siRNA** | | **Sequences (5’-3’)** |
| --- | --- | --- |
| MYBL2#1 | sense | CUGGCAUCGAACUCAUCAU dTdT |
|  | antisense | AUGAUGAGUUCGAUGCCAG dTdT |
| MYBL2#3 | sense | CUGGAACUCUACCAUCAAA dTdT |
|  | antisense | UUUGAUGGUAGAGUUCCAG dTdT |
| CCL2 | sense | GCUGUUAUAACUUCACCAAUA dTdT |
|  | antisense | UAUUGGUGAAGUUAUAACAGC dTdT |
| CCL20 | sense | GCAAGCAACUUUGACUGCUGUCUUG dTdT |
|  | antisense | CAAGACAGCAGUCAAAGUUGCUUGC dTdT |
| CXCL5 | sense | CUGAAGAACGGGAAGGAAA dTdT |
|  | antisense | UUUCCUUCCCGUUCUUCAG dTdT |
| CXCL8 | sense | AAAGCUUUACAAUAAUUUCUG dTdT |
|  | antisense | CAGAAAUUAUUGUAAAGCUUU dTdT |

**Supplementary Figures**

Supplementary Figure1


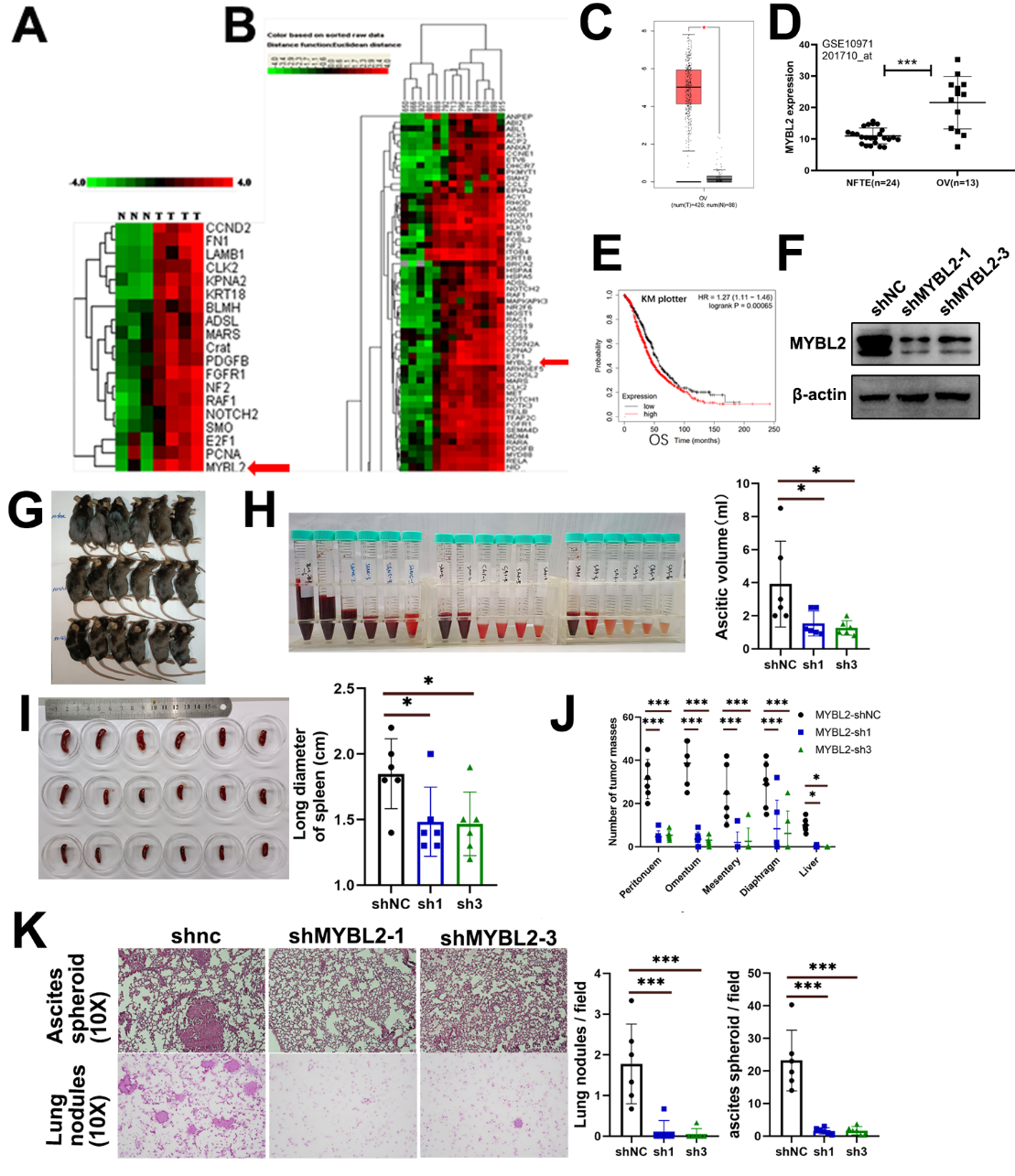


Supplementary Figure2


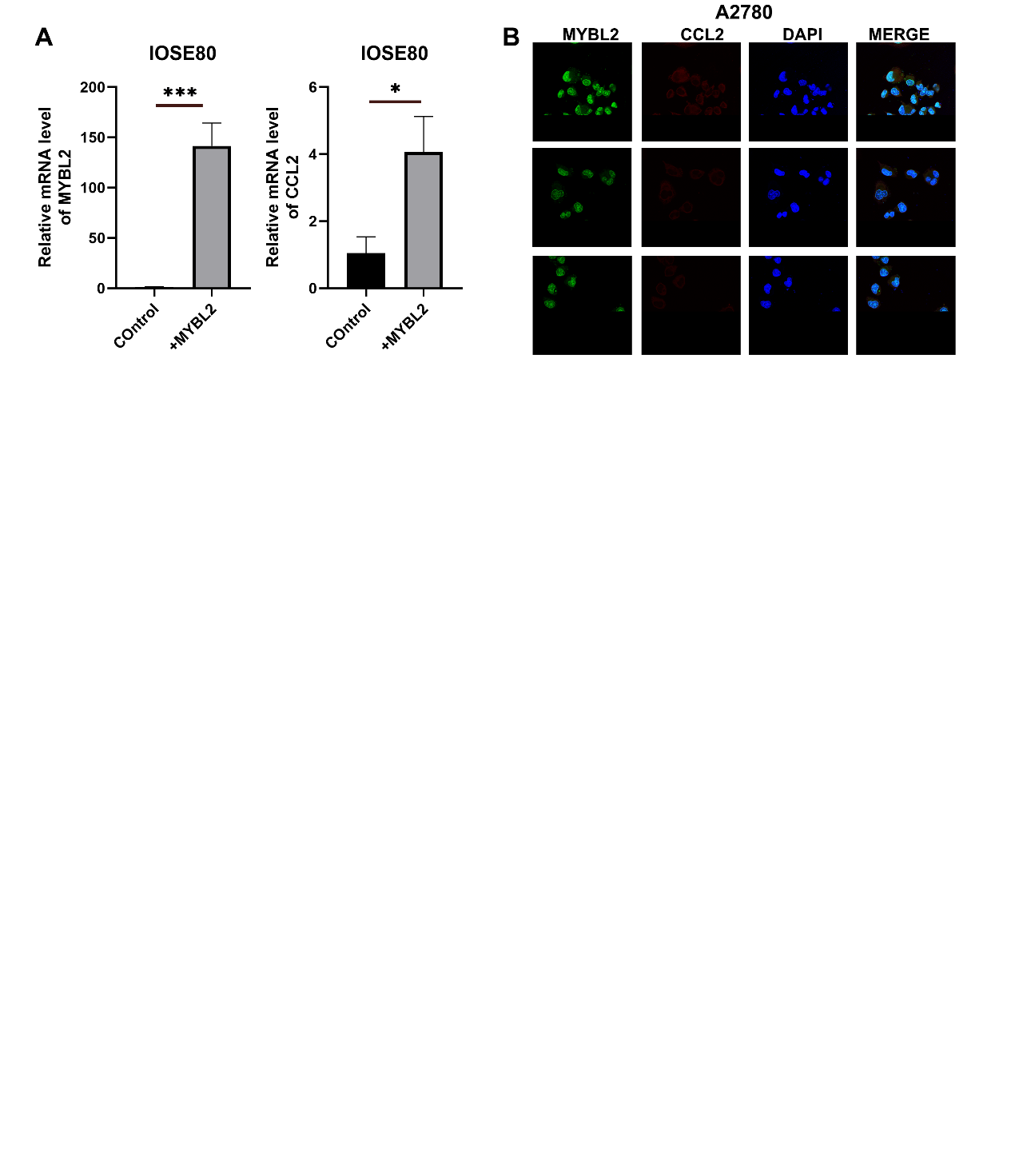


Supplementary Figure3


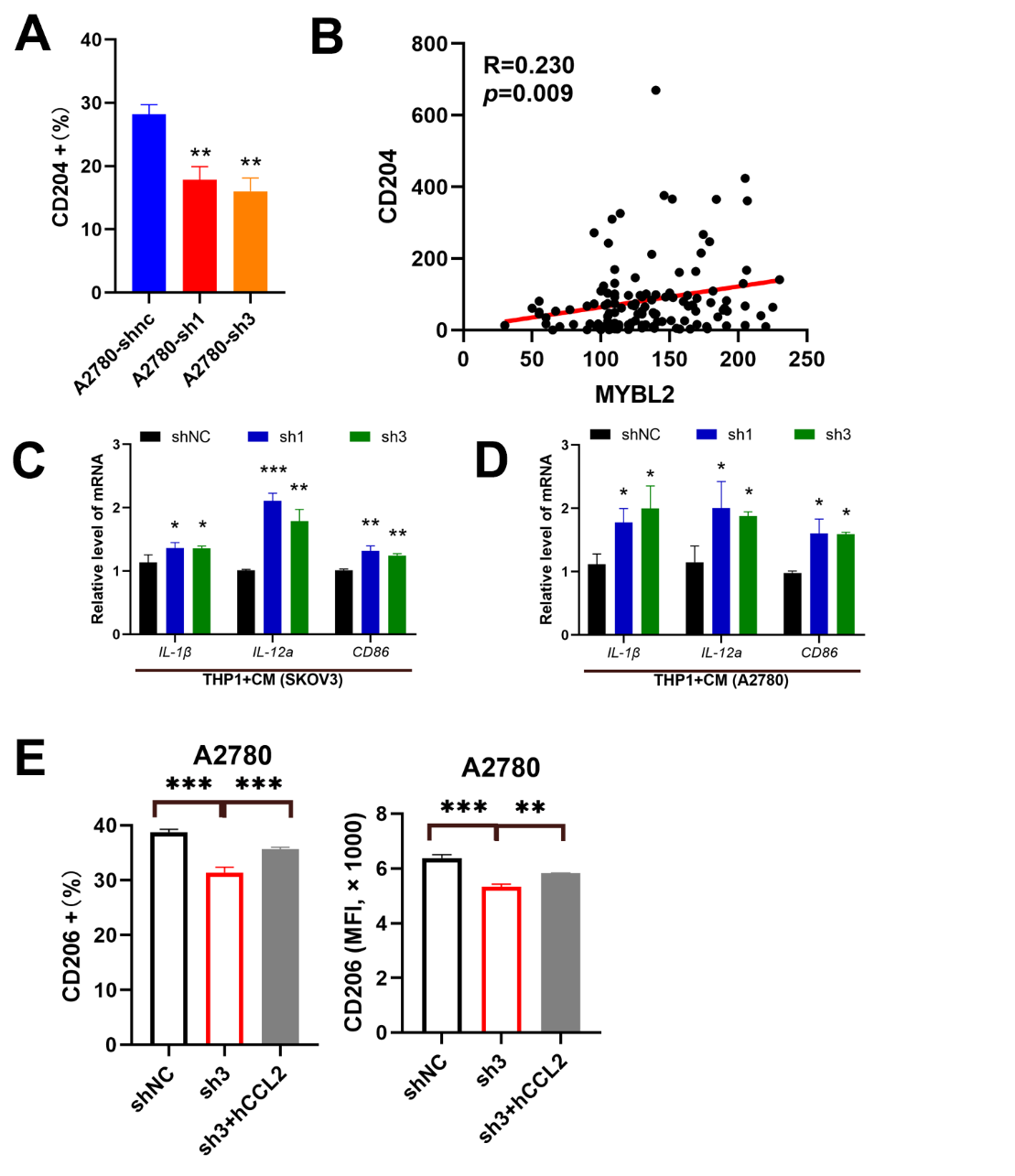


Supplementary Figure4


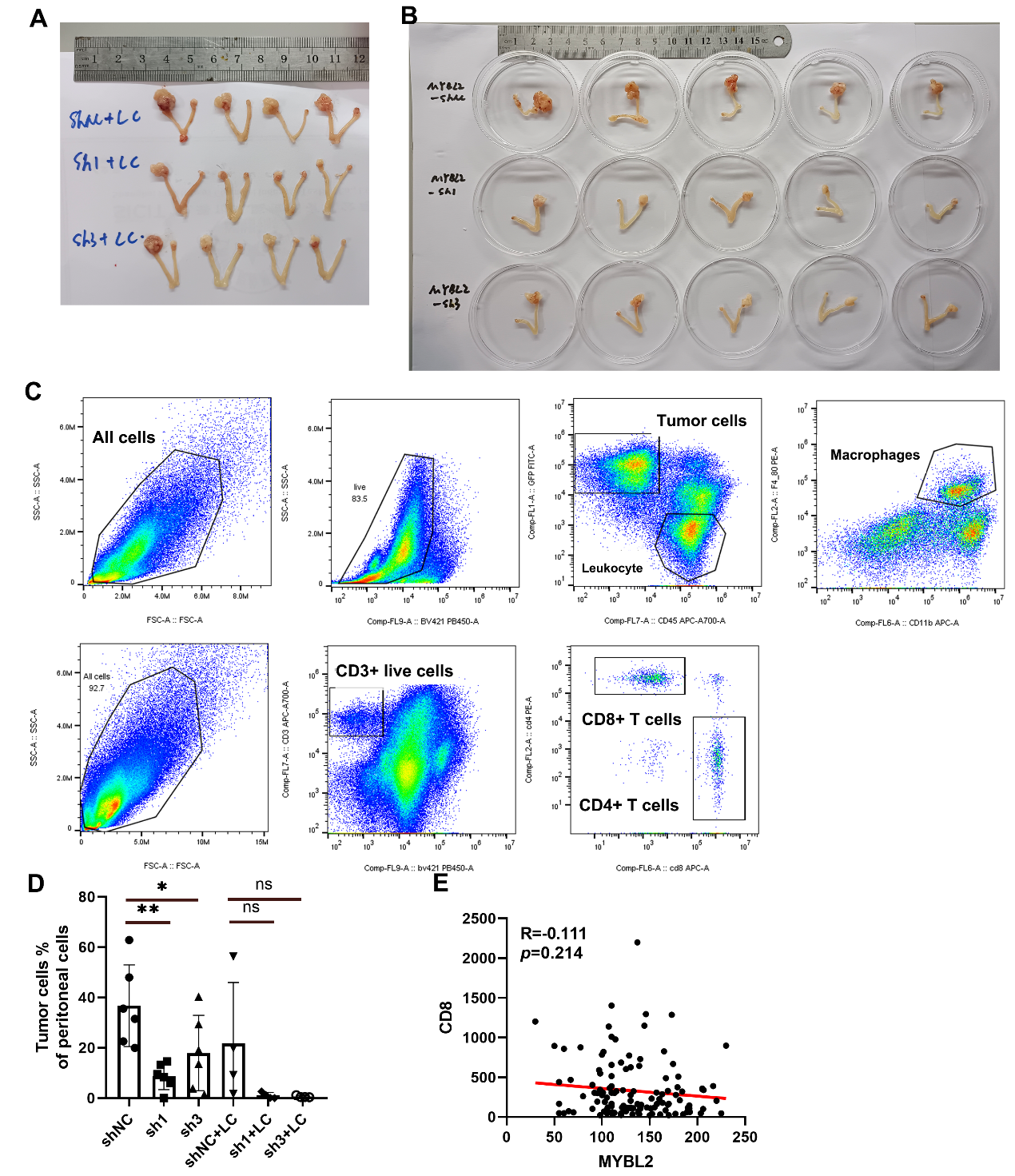


Supplementary Figure5


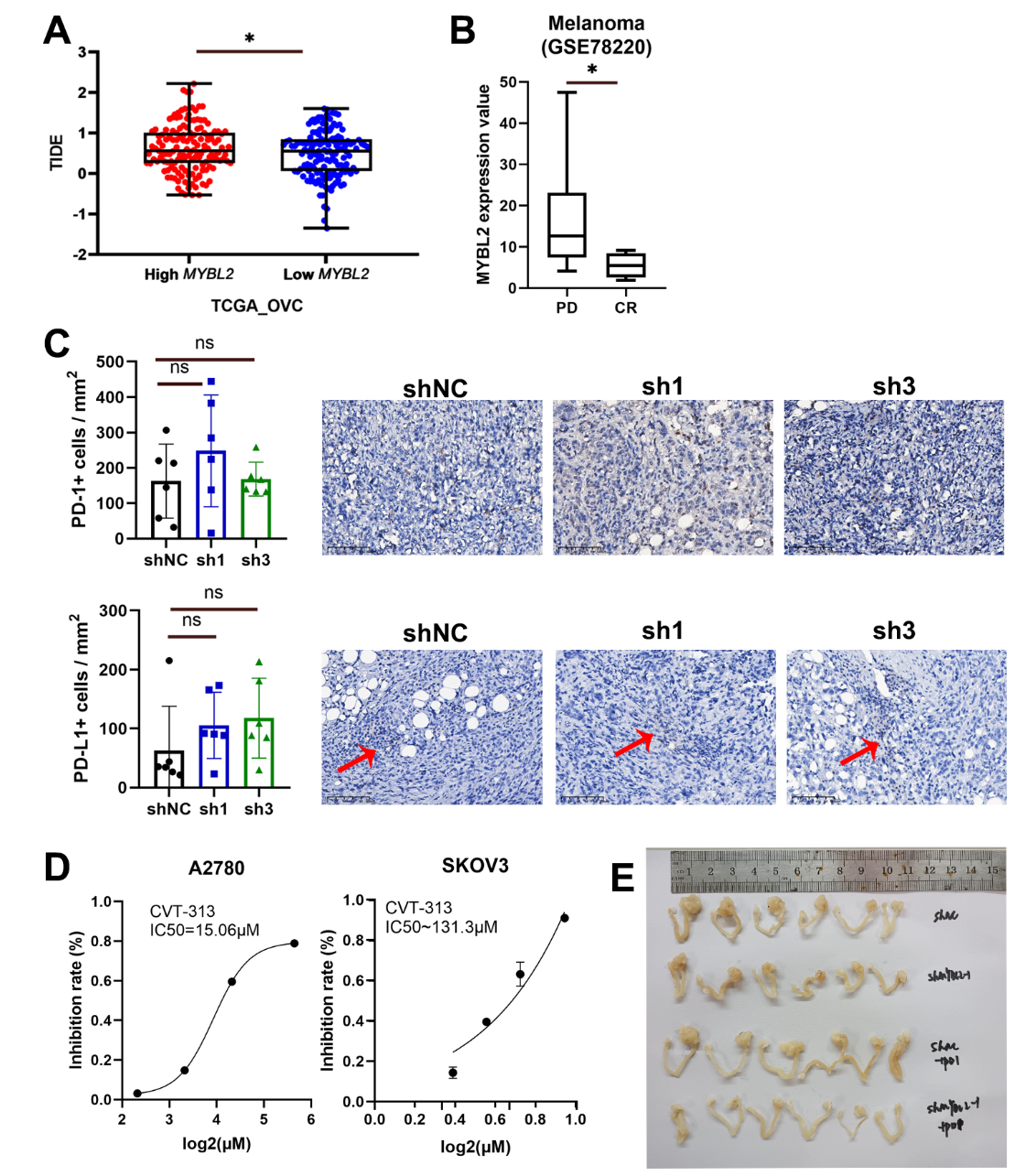


Original raw western blot data


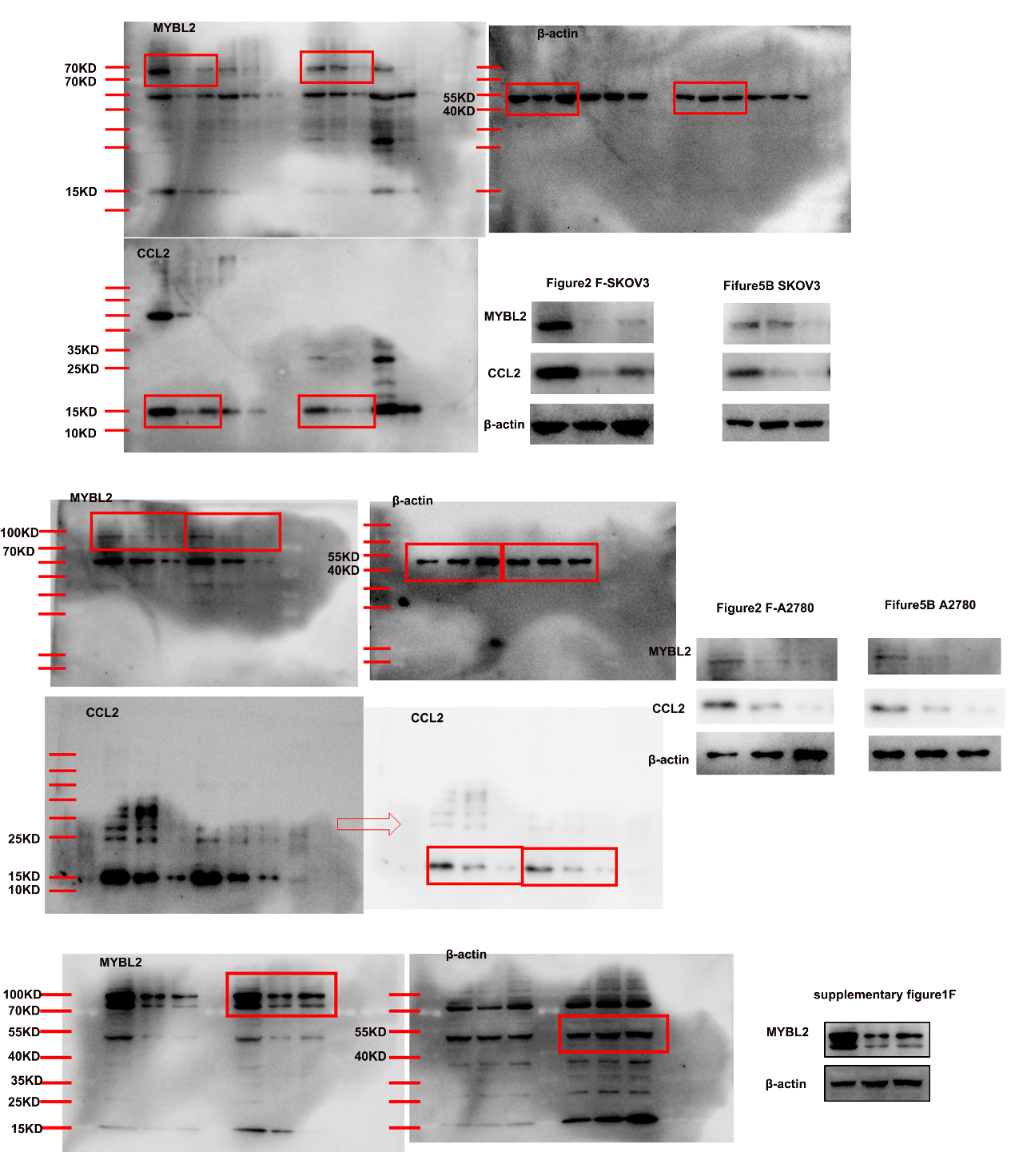

Supplement: Supplementary file 1 — Supplementary Material 1 [file 12935_2023_3079_MOESM1_ESM.docx]
